# Electronic patient-reported outcomes in clinical kidney practice (ePRO Kidney): a process evaluation of educational support for clinicians

**DOI:** 10.1177/20406223231173624

**Published:** 2023-06-12

**Authors:** Kara Schick-Makaroff, Scott Klarenbach, Jae-Yung Kwon, S. Robin Cohen, Joanna Czupryn, Loretta Lee, Robert Pauly, Jennifer M. MacRae, Bruce Forde, Richard Sawatzky

**Affiliations:** Faculty of Nursing, University of Alberta, 4-116 Edmonton Clinic Health Academy, 11405-87 Avenue, Edmonton, AB T6G 1C9, Canada; Division of Nephrology, Faculty of Medicine & Dentistry, University of Alberta, Edmonton, AB, Canada; School of Nursing, University of Victoria, Victoria, BC, Canada; Departments of Oncology and Medicine, McGill University, Montreal, QC, Canada; Lady Davis Research Institute of the Jewish General Hospital, Montreal, QC, Canada; Faculty of Nursing, University of Alberta, Edmonton, AB, Canada; Faculty of Nursing, University of Alberta, Edmonton, AB, Canada; Division of Nephrology, Faculty of Medicine & Dentistry, University of Alberta, Edmonton, AB, Canada; Cumming School of Medicine, University of Calgary, Calgary, AB, Canada; Cambian Business Services, Surrey, BC, Canada; School of Nursing, Trinity Western University, Langley, BC, Canada; Centre for Health Evaluation & Outcome Sciences, St. Paul’s Hospital, Vancouver, BC, Canada; Sahlgrenska Academy, University of Gothenburg, Gothenburg, Sweden

**Keywords:** electronic patient-reported outcome, health services, home hemodialysis, peritoneal dialysis, person-centered care

## Abstract

**Background:**

Patient-reported outcomes (PROs) are increasingly mandated in kidney care to incorporate patients’ perspectives.

**Objectives:**

We assessed whether educational support for clinicians using electronic (e)PROs could enhance person-centered care.

**Design:**

A process evaluation, using a mixed methods longitudinal comparative concurrent design was undertaken of educational support to clinicians on routine use of ePROs. In two urban home dialysis clinics in Alberta, Canada, patients completed ePROs. At the implementation site, clinicians were provided with ePROs and clinician-oriented education via voluntary workshops. At the non-implementation site, neither were provided. Person-centered care was measured using the Patient Assessment of Chronic Illness Care-20 (PACIC-20).

**Methods:**

Longitudinal structural equation models (SEMs) compared change in overall PACIC scores. The interpretive description approach, using thematic analysis of qualitative data, further evaluated processes of implementation.

**Results:**

Data were collected from questionnaires completed by 543 patients, 4 workshops, 15 focus groups, and 37 interviews. There was no overall difference in person-centered care throughout the study, including after delivery of workshops. The longitudinal SEMs revealed substantial individual-level variability in overall PACIC trajectories. However, there was no improvement at the implementation site and no difference between the sites during both the pre- and post-workshop periods. Similar results were obtained for each PACIC domain. Qualitative analysis provided insights into why there was no substantial difference between sites: (1) clinicians wanted to see kidney symptoms, not quality of life, (2) workshops were tailored to clinicians’ educational needs, not patients’ needs, and (3) variable use of ePRO data by clinicians.

**Conclusion:**

Training clinicians on use of ePROs is complex and likely only part of what is required to enhance person-centered care.

**Registration:**

NCT03149328. https://clinicaltrials.gov/ct2/show/NCT03149328

## Introduction

Internationally, patient-reported outcomes (PROs) are being collected, at times mandated, in kidney care.^1,2^ PRO measures are instruments that patients complete to self-report health outcomes relevant to their quality of life (QOL).^3^ They are systematically collected with the hope that patients’ responses will inform and guide their care,^1,4–8^ and in turn support person-centered kidney care.^4,9–13^ The global kidney community acknowledges the urgent need for evidence-informed strategies to improve both the QOL and healthcare experiences of people living with dialysis.^14^ Optimal use of PROs at point of care may help address this need.

Use of PRO data to enhance person-centered dialysis care remains largely unexplored,^1^ perhaps because routine collection, use, and integration is a complex intervention. Person-centered dialysis care upholds attributes of centeredness, namely that a patient is a person who is unique, and has the right to be heard and to have shared responsibility of their care.^15^ Use of PROs is one way of drawing attention to the patients’ perspectives of their health outcomes. Decades of research has previously focused on efficiency or effectiveness of PRO data usage to support monitoring symptoms or communication in various populations,^16^ including kidney care settings.^1,17,18^ However, international evidence-based sources and syntheses have identified that clinicians need training and education to support PRO use in their clinical practice at point of care^2,19–23^ (i.e. at the individual level between a person and clinician/s) to enhance person-centered care.

While multidisciplinary kidney practitioners have expressed some support for use of PRO responses at point of care,^6,17,24^ they have also identified barriers. For example, they expressed skepticism about the benefits to routine patient care,^24^ confused/conflated clinician assessment with PRO self-report,^24,25^ perceived responses to address concerns are beyond their scope of practice,^7,26^ lacked effective interventions to address concerns,^7,26^ worried that PRO collection might increase their workload^6,8,24^ and psychological referrals,^24^ and feared litigation of negligence if PRO responses were not addressed.^24^ These uncertainties have resulted in poor uptake, further identifying the need for a clinician-oriented intervention.

Understanding how to provide educational support to clinicians to optimize use and integration of kidney patients’ PRO responses is crucial. The purpose of the study was to assess, from clinicians’ and patients’ points of view, how person-centered care could be enhanced through clinician-oriented educational support for multidisciplinary kidney clinicians in routinely utilizing electronic PROs (ePROs). The research question was: To what extent do clinicians and patients report improved person-centered kidney care associated with educational support to clinicians on use of ePROs?

## Methods

### Design, complex intervention, sites

This mixed methods study was a process evaluation of educational support to clinicians on routine use of ePROs, which is a complex intervention.^27^ We used a longitudinal comparative concurrent design,^28^ with clinician and patient focus groups and interviews. The rationale for this approach was to provide contextual understandings and practice implications of the impact of the complex intervention: clinician-oriented educational support for routine use of ePROs. The study was registered.^29^

**Figure 1.**
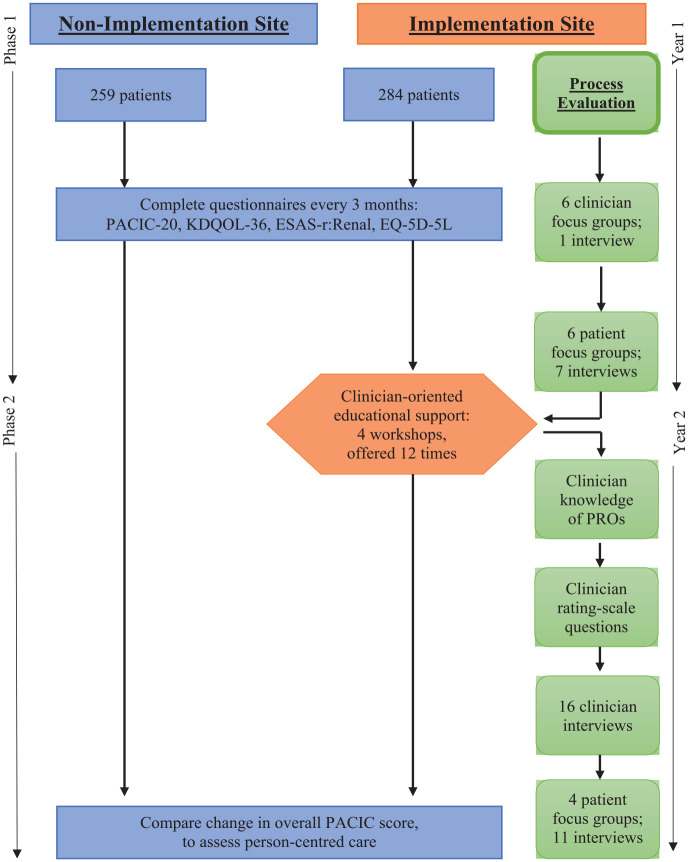
Project timeline.

There were two phases over 2 years at two sites. See Figure 1 – Project timeline. The sites included two home dialysis clinics (peritoneal dialysis and home hemodialysis) providing care to all of Alberta, Canada: Northern (implementation site) and Southern (non-implementation site). The two sites were comparable in geographic service to Albertans, dialysis staffing, facilities, and number of home hemodialysis patients (<100); however, the implementation site had more peritoneal dialysis patients overall (>300) than the non-implementation site (>200). At the implementation site, ePRO responses were provided to clinicians; they had collected PROs on paper for 8 years prior to the study. In the non-implementation site, ePRO responses were not provided to clinicians; patients had not previously been invited to complete PROs. In both the sites, patients had online access to their ePRO data, to track responses overtime, share with any of their healthcare team, or use in self-care.

**Figure 2.**
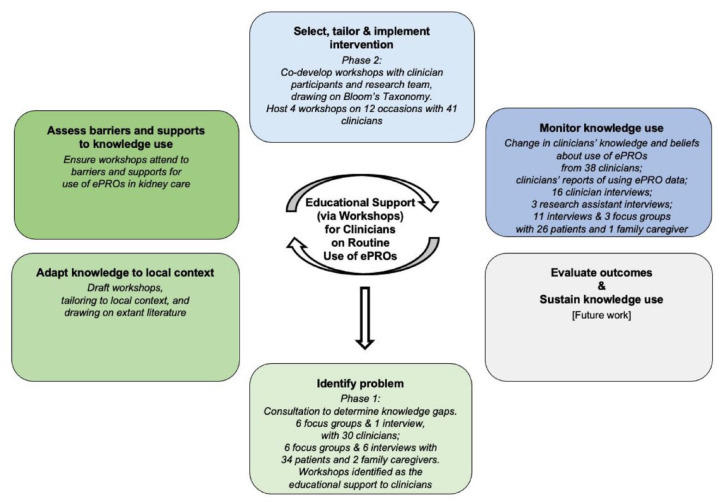
Adapted knowledge-to-action framework. Graham and Tetroe.^30^

The Knowledge-To-Action Framework^30^ guided both phases of this patient-oriented research.^31^ See Figure 2 – Adapted Knowledge-to-Action Framework. Phase I included clinician and patient consultation at the implementation site to develop the clinician-oriented educational support. Following the knowledge-to-action steps, in consultation to determine knowledge gaps of routine use of ePROs, the education support that clinicians endorsed were tailored, co-designed workshops. Workshops included knowledge adapted to the local context, and content that addressed barriers and supports to use of ePROs in kidney care. In phase II, the implementation site was supported via clinician workshops.

Our research team encompassed multi-disciplinary clinicians, decision-makers, researchers, patient organizations, and patients. The Patient Advisory Committee was co-chaired by LL, a Patient Partner, and Advisory members were trained in patient-oriented research and involved throughout the project.

### Participant recruitment

Through purposive sampling,^32^ all clinicians (nurses, nephrologists, dietitians, social workers) from the implementation site were invited via information sessions, posters, and emails from the department between July 2017 and July 2019. Patients attending their regularly scheduled clinic appointments at both sites were initially screened and invited to participate by a unit clerk or a nurse, and then followed up by study staff present in waiting rooms. Patients were eligible if they were age ⩾18, could read/write in English, and provide written consent; and ineligible if they had visual impairment, severe cognitive impairment, or were in health crisis. At the implementation site, patients were invited to participate in focus groups or interviews. Although caregivers were not recruited, they were admitted if required to support the patient. Recruitment of both clinicians and patients was ongoing throughout study through purposive and snowball sampling.^32^ Ethical approval was granted by University of Alberta/Alberta Health Services (#Pro00068365) and the University of Calgary (#REB17-0506). Informed consent was obtained at the first point of data collection, re-confirmed at subsequent interactions.

### Data collection and analysis

#### Quantitative measures

Throughout phases I and II, patients at both sites completed all questionnaires by accessing a digital health platform (Cambian).^33^ Research staff phoned/emailed/mailed patients 7–14 days prior to appointments (scheduled approximately every 3 months), inviting them to complete questionnaires online, or via phone or mail if needed. The Patient Assessment of Care for Chronic Conditions-20 questionnaire (PACIC-20)^34^ was used to assess person-centered care. Other ePRO questionnaires were as follows: (1) Edmonton Symptom Assessment System-renal (ESAS-r:Renal),^35^ (2) Kidney Disease Quality of Life-36 (KDQOL-36),^36^ (3) EQ-5D-5L^37^ (see Table 1). Internal consistency reliability (based on the ordinal Cronbach’s alpha)^38^ in this sample was 0.97 for the overall PACIC score, and ranged from 0.82 (delivery system design) to 0.91 (problem-solving) for the domain scores.

**Table 1.** ePRO data collection.

Instrument	Construct being measured	No. of items and response scale(s)	Domains measured
Patient Assessment of Care for Chronic Conditions (PACIC 20)	Person-centered care	20 items with 5-point response scale (from ‘almost never’ to ‘almost always’) Recall: past 6 months Range of scores 1–5 Higher scores indicate perception of greater involvement in self-management and greater receipt of chronic care counseling	Five sub-scales: Patient activation, Delivery system design, Goal setting, Problem-solving, Follow-up
Edmonton Symptom Assessment System-revised for Renal (ESAS-r:Renal)	Symptoms	12 items with a response scale ranging from 0 (no symptom) to 10 (worst possible) Recall: past week Items range 0–10 Items are not added into a total score Higher scores indicate worse symptoms/well-being	11 items measure individual symptoms, 1 measures well-being
Kidney Disease Quality of Life-36 (KDQOL-36)	Quality of life	36 items with 2-, 3-, 5-, and 6-point response scales Recall: past four weeks Each domain is transformed to a 0–100 possible score range Higher scores indicate better quality of life	Five scales: Symptoms and problems, Effects of kidney disease, Burden of kidney disease, Summary scales (derived from SF-12) include: Physical component summary, Mental component scale
EQ-5D-5L	Health utility	Five dimensions and each dimension has five levels of severity Range of scores for each level is 1–5 Higher scores indicate worse health state Visual analog scale (VAS) of ‘how good or bad your health is today’ from 0 (worst) to 100 (best) Higher scores indicate worse health state Recall: describe your health today	Five dimensions: Mobility, Self-care, Usual activities, Pain or discomfort, Anxiety or depression

Completed by patients at each site during regularly scheduled clinic appointments.

ePRO, electronic patient-reported outcome.

In phase II, clinicians in the implementation site were asked to anonymously answer two rating-scale questions asking how often they reviewed and used ePRO data to inform their care. Clinicians were asked to respond to eight questions about their knowledge and beliefs regarding the use of ePROs prior to the first workshop and after all workshops were completed.

#### Quantitative analysis

We used descriptive statistics to compare the samples at the two sites at the start of the study (time 0) using the Chi-square test for categorical variables and *t*-test for continuous variables.

To address the research question, we first graphically compared the average linear changes over time for the overall PACIC score and domain scores between the two sites over the 24-month period. We subsequently fit longitudinal structural equation models (SEMs), using Mplus software (version 8.6),^39^ to statistically compare average change over time between the two sites. Separate SEMs were fit before and after the workshop start date. The SEMs were specified with two latent factors representing the intercept (outcome score at start of study) and the slope (linear change in outcome scores) over eight repeated measures that occurred at the time of each visit, which were regressed on the grouping variable (site) and the following covariates to control for statistically significant differences between sites: education, ethnic group, and diabetes. Individually varying times of observations relative to the start of study were specified to account for variations in study entry and the timing of each visit. Multilevel multiple imputation with covariates^40^ was applied to created 20 data files with imputed item response data for participants who had missing data for one or more of the PACIC items (5.0% imputed data). Full information maximum likelihood was applied to accommodate unequal numbers of measurement occasions for each person.

#### Qualitative data

Qualitative data were collected across both phases in the implementation site. Data were collected via 12 focus groups (6 clinician, 6 patient) and 7 interviews (1 clinician, 6 patients) during phase I (July 2017–May 2018), and via 30 interviews (16 clinicians, 11 patients, 3 research assistants) and 3 patient focus groups during phase II (February 2019–August 2019). Interviews or focus groups were conducted by the study lead (KS-M), project coordinator (JC), and two trainees (nursing students) in private rooms at the clinic, at the university, or by phone, lasting 30–90 min. There were no prior relationships between interviewers and participants. Regular debriefing between interviewers and study leads enhanced data quality and reflexivity. Semi-structured interview or focus group guides (Supplementary File 1) were iteratively revised with the research team and Patient Advisory Committee, informed by ongoing data analysis. Interviews or focus groups were audio-recorded and transcribed verbatim.

#### Qualitative analysis

Guided by the interpretive description approach,^41^ data were analyzed in phase I to guide ePRO provision and inform workshop creation, and in phase II to assess how person-centered care was enhanced (or not) through routine ePRO use. Transcripts from initial interviews or focus groups were listened to and read to create a codebook, iteratively refined during analysis. The data were coded by JC and a trainee under the supervision of KS-M; questions about categorization were resolved through consensus. Managed with N-Vivo^TM^, data were thematically analyzed for meaning saturation^42,43^ to understand diversity of perspectives. Patient and clinician data were analyzed separately, then compared and contrasted. We ensured trustworthiness and rigor by addressing credibility (iterative discussions, triangulation, and negative/alternative case analysis), confirmability (audit trail, field notes), and transferability (context).^41^ The Consolidated Criteria for Reporting Qualitative Research guidelines^44^ in Supplementary File 2 provides additional details.

## Results

### Participant demographics

Of the 693 approached patients, 543 (78%) participated and routinely provided ePROs (Table 2). Patients had a mean age of 56, identified primarily as Caucasian (65.4%), were predominantly male (65.8%), received peritoneal dialysis (71.3%); 56 withdrawals; 41 loss-to-follow-up. In total, 2252 ePROs were completed over 2 years. At the implementation site, 198 patients chose to complete online; 86 by phone or mail. At the non-implementation site, 111 chose online; 148 by phone or mail. Interviews and focus groups were attended by 42 patients, 3 caregivers, and 34 clinicians. Forty-one clinicians attended between one and four workshops, and 38 completed questionnaires before and after workshops (Table 3).

**Table 2.** Characteristics of patients by site at baseline.

Characteristics	Total (*N* = 543, 100%)	Implementation (*N* = 284, 52.3%)	Non-implementation (*N* = 259, 47.7%)	*p*-value
Age (*n* = 541), mean (SD)	56.0 (13.8)	55.4 (13.3)	56.7 (14.2)	0.25
Gender (*n* = 541)				0.99
Female	34.2	34.4	34.0	
Male	65.8	65.6	66.0	
Highest level of education (*n* = 541)				0.02
Elementary school	4.3	6.3	1.9	
High school graduate	29.9	33.5	26.1	
College, trade, diploma	39.6	35.6	44.0	
Undergraduate degree	16.6	14.1	19.5	
Post-graduate degree	7.9	8.8	7.0	
Other	1.7	1.8	1.6	
Employment status (*n* = 541)				0.10
Retired	31.8	27.2	36.8	
Unable to work	26.4	29.0	23.6	
Working	30.5	31.4	29.5	
Other	11.3	12.4	10.1	
Ethnicity (*n* = 543)				0.001
Aboriginal	5.9	8.1	3.5	
Asian	19.2	14.8	23.9	
White (Caucasian)	65.4	69.0	61.4	
Black	2.6	1.4	3.9	
Other	3.7	4.9	2.3	
More than one race or ethnicity	3.1	1.8	4.6	
Unknown or not reported	0.2	0	0.4	
Type of dialysis (*n* = 541)				0.10
Peritoneal	71.3	70.6	72.2	
Home hemodialysis	25.9	26.6	25.1	
Nocturnal	2.0	2.1	1.9	
Other	0.7	0.7	0.8	
Diagnoses (*n* = 543)				
Diabetes (Y/N)^a^	36.6	40.8	32.0	0.04
Hypertension (Y/N)^a^	67.8	64.1	71.8	0.07
Myocardial infarction (Y/N)^a^	9.8	9.5	10.0	0.95
Heart disease (Y/N)^a^	12.0	13.7	10.0	0.23
Stroke (Y/N)^a^	4.8	5.6	3.9	0.44
Leg amputation (Y/N)^a^	3.1	3.5	2.7	0.76
Lung disease (Y/N)^a^	4.2	2.8	5.8	0.13
Liver disease (Y/N)^a^	1.5	0.7	2.3	0.23
Arthritis (Y/N)^a^	16.0	13.4	18.9	0.10
Cancer (Y/N)^a^	6.4	7.7	5.0	0.26
Lower back pain (Y/N)^a^	15.3	15.1	15.4	1.00
Depression(Y/N)^a^	12.3	12.7	12.0	0.90
Other (Y/N)^a^	11.2	9.9	12.7	0.35
ePROs at baseline				
Pain, mean (SD) (*n* = 533)	2.8 (2.7)	2.6 (0.2)	2.8 (0.2)	−0.003
Tiredness, mean (SD) (*n* = 533)	4.7 (2.6)	2.5 (0.1)	2.8 (0.2)	−0.383
Drowsiness, mean (SD) (*n* = 526)	3.6 (2.8)	2.6 (0.2)	2.9 (0.2)	−0.152
Nausea, mean (SD) (*n* = 528)	1.5 (2.3)	2.4 (0.1)	2.1 (0.1)	0.204
Appetite, mean (SD) (*n* = 529)	2.5 (2.8)	2.6 (0.2)	2.9 (0.2)	−0.211
Shortness of breath, mean (SD) (*n* = 532)	2.2 (2.6)	2.4 (0.1)	2.7 (0.2)	0.128
Depression, mean (SD) (*n* = 532)	2.1 (2.6)	2.5 (0.2)	2.6 (0.2)	−0.071
Anxiety, mean (SD) (*n* = 532)	2.0 (2.5)	2.5 (0.1)	2.6 (0.2)	0.017
Wellbeing, mean (SD) (*n* = 527)	3.7 (2.5)	2.5 (0.1)	2.6 (0.2)	−0.186
Itching, mean (SD) (*n* = 525)	3.0 (3.0)	2.8 (0.2)	3.2 (0.2)	−0.253
Sleeping, mean (SD) (*n* = 531)	4.1 (3.1)	3.1 (0.2)	3.2 (0.2)	−0.27
Restless legs, mean (SD) (*n* = 526)	2.8 (3.0)	3.0 (0.2)	3.1 (0.2)	−0.07
PCS, mean (SD) (*n* = 443)	36.5 (9.8)	38.2 (9.9)	35.1 (9.6)	< 0.001
MCS, mean (SD) (*n* = 443)	48.3 (10.4)	48.5 (10.2)	48.1 (10.5)	0.699
Burden of kidney disease, mean (SD) (*n* = 479)	42.5 (26.1)	43.6 (26.5)	41.6 (25.7)	0.399
Symptoms/problems, mean (SD) (*n* = 480)	76.2 (14.8)	78.1 (14.3)	74.5 (15.1)	0.008
Effects of kidney disease, mean (SD) (*n* = 480)	65.7 (21.3)	67.7 (20.9)	64.0 (21.5)	0.058
PACIC, mean (SD) (*n* = 473)	3.5 (0.9)	3.6 (1)	3.5 (0.9)	0.232
EQ-5D-5L, mean (SD) (*n* = 470)	0.8 (0.2)	0.8 (0.2)	0.7 (0.2)	< 0.001

ePRO, electronic patient-reported outcome; eQ-5D-5L, health equity; MCS, mental component summary; PACIC, Patient Assessment of Chronic Illness Care; PCS, physical component summary; SD, standard deviation.

a Each variable treated as a separate variable.

**Table 3.** Demographics of participants in qualitative focus groups and interviews, and clinician workshops.

Demographic information	Qualitative focus groups and interviews			Clinician workshops^a^	
	Patients	Caregivers	Healthcare providers	Physician	Allied health
Total unique participants	42	3	34	14	24
Phase I (phase II)^b^	34 (26)	2 (1)	30 (16)	N/A	N/A
Gender					
Female	13 (12)	1 (1)	19 (10)	3	20
Male	21 (14)	1 (0)	11 (6)	11	4
Age					
Mean age (years)	53.1 (55.5)	66.5 (68)	42.8 (42.3)	41.7	42.6
Range (min–max)	28–79 (35–74)	66–67 (68)	26–57 (26–65)	27–65	26–59
Highest level of education					
Elementary	2 (1)	0 (0)	0 (0)	N/A	N/A
High School	8 (6)	1 (0)	0 (0)	N/A	N/A
College	14 (9)	0 (0)	5 (4)	0	9
Undergraduate degree	5 (3)	1 (1)	10 (6)	0	12
Postgraduate degree	4 (6)	0 (0)	12 (3)	12	2
Pre-Med	1 (1)	0 (0)	0 (0)	N/A	N/A
BSc	0 (0)	0 (0)	1 (0)	0	0
PhD	0 (0)	0 (0)	1 (1)	1	0
Professional degree	0 (0)	0 (0)	1 (1)	1	0
Nursing	0 (0)	0 (0)	0 (1)	0	1
Employment status					
Permanent	N/A	N/A	N/A	10	13
Part time	5 (5)	0 (0)	4 (2)	0	5
Full time	5 (5)	0 (0)	23 (12)	N/A	N/A
Casual	0 (0)	0 (0)	1 (0)	0	2
Temporary full time	0 (0)	0 (0)	1 (1)	N/A	N/A
Administrative	0 (0)	0 (0)	0 (1)	1	1
Trainee	N/A	N/A	N/A	2	0
Resident	0 (0)	0 (0)	1 (0)	1	0
Retired	10 (7)	1 (1)	0 (0)	N/A	N/A
Cannot work	12 (6)	0 (0)	0 (0)	N/A	N/A
Unemployed	1 (2)	0 (0)	0 (0)	N/A	N/A
Self-employed	1 (1)	1 (0)	0 (0)	N/A	N/A
Missing	N/A	N/A	N/A	0	3
Occupation					
Nephrologist	N/A	N/A	11 (5)	14	0
Nurse	N/A	N/A	17 (9)	0	19
Social Worker	N/A	N/A	1 (1)	0	2
Administrative	N/A	N/A	N/A	0	1
Dietitian	N /A	N/A	0 (1)	0	1
Unit Manager	N/A	N/A	1 (0)	0	1
Type of dialysis^c^					
Peritoneal	20 (13)	2 (0)^d^	13 (7)	0	15
Home hemodialysis	13 (12)	0 (1)^d^	6 (3)	0	6
Nocturnal	1 (1)	0 (0)	N/A	N/A	N/A
Peritoneal and Home Hemodialysis	N/A	N/A	11 (6)	14	3
Ethnic background					
Caucasian	26 (21)	2 (1)	N/A	N/A	N/A
South Asian	2 (2)	0 (0)	N/A	N/A	N/A
Chinese	1 (0)	0 (0)	N/A	N/A	N/A
Latin American	1 (0)	0 (0)	N/A	N/A	N/A
Aboriginal	3 (2)	0 (0)	N/A	N/A	N/A
African	0 (1)	0 (0)	N/A	N/A	N/A
Cajun	1 (0)	0 (0)	N/A	N/A	N/A

N/A, not applicable.

a Clinicians that attended one or more workshops and responded to eight questions about their knowledge and beliefs regarding the use of patient-reported outcomes (PROs) prior to the first workshop and after all workshops were completed.

b Phase II is denoted in round brackets. Note that there were eight participants in phase II who were not a part of phase I (eight patients), and 30 participants who were in phase I but not a part of phase II (16 patients and 14 healthcare providers).

c For clinicians, type of dialysis in their practice.

d Caregiver to a person with this modality.

### Person-centered care

**Figure 3.**
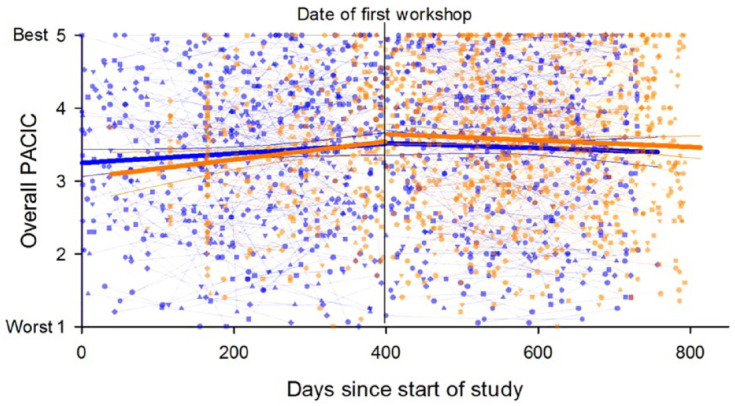
Trajectories of PACIC by site pre- and post-workshops. Blue represents implementation site. Orange represents non-implementation site. Different shapes represent individual-level data points for observed data (no imputation). Bold lines represents average linear trajectories without adjustment for covariates. PACIC, Patient Assessment of Chronic Illness Care.

Figure 3 depicts substantial individual-level variability in overall PACIC trajectories, with greater improvement over time in the non-implementation site relative to the implementation site during the pre-workshop period, whereas the trajectories appear more similar during the workshop period. The results of the SEMs (see Table 4) confirmed that the overall PACIC score was lower at the start of the study for the non-implementation site (intercept of non-implementation = 3.10, *p* = 0.00; difference in intercepts = 0.32 for the implementation site, *p* = 0.02) with greater improvement over time during the pre-workshop period (difference in slopes = −0.41, *p* = 0.02, for the implementation site). However, there was no detectable change over time in overall PACIC scores during the post-workshop periods in both sites (slope of non-implementation = −0.08, *p* = 0.27; difference in slopes = −0.03, *p* = 0.80). The SEM results for PACIC domains suggest that the intercepts and slopes were very similar between the two sites pre- and post-workshops for most of the domains; the only detected differences were for the pre-workshop intercepts and slopes for goal setting (difference in intercepts = 0.40, *p* = 0.03; difference in slopes = −0.46, *p* = 0.05) and for follow-up (difference in intercepts = 0.52, *p* = 0.01; difference in slopes = −0.64, *p* = 0.01).

**Table 4.** Structural equation modeling results.

Measure^a^	Pre-^b^ / Post-workshop	Regression of intercept		Regression of slope		Model fit	
		Intercept (*p-*value)	Site^c^ (*p-*value)	Slope (*p-*value)	Site^c^ (*p-*value)	LL	BIC
Overall PACIC	Pre	3.10 (0.00)	0.32 (0.02)	0.32 (0.05)	−0.41 (0.02)	−1045.17	2266.53
	Post	3.60 (0.00)	−0.04 (0.85)	−0.08 (0.27)	−0.03 (0.80)	−1416.53	3035.03
Patient activation	Pre	3.45 (0.00)	0.15 (0.49)	0.18 (0.39)	−0.23 (0.36)	−1363.93	2904.05
	Post	3.67 (0.00)	0.13 (0.65)	−0.02 (0.84)	−0.14 (0.41)	−1812.13	3826.22
Delivery system design	Pre	3.49 (0.00)	0.27 (0.10)	0.22 (0.24)	−0.28 (0.19)	−1212.69	2601.56
	Post	4.10 (0.00)	−0.30 (0.24)	−0.21 (0.04)	0.15 (0.34)	−1649.71	3501.38
Goal setting	Pre	2.84 (0.00)	0.40 (0.03)	0.40 (0.06)	−0.46 (0.05)	−1271.60	2719.38
	Post	3.37 (0.00)	0.06 (0.85)	−0.00 (0.99)	−0.12 (0.52)	−1745.13	3692.22
Problem-solving	Pre	3.61 (0.00)	0.20 (0.28)	0.20 (0.32)	−0.36 (0.11)	−1302.03	2780.24
	Post	4.04 (0.00)	−0.01 (0.96)	−0.15 (0.08)	−0.06 (0.68)	−1734.24	3670.45
Follow-up	Pre	2.80 (0.00)	0.52 (0.01)	0.54 (0.01)	−0.64 (0.01)	−1282.83	2741.84
	Post	3.67 (0.00)	−0.26 (0.33)	−0.16 (0.07)	0.10 (0.54)	−1704.56	3611.10

BIC, Bayesian Information Criterion; LL, log-likelihood; PACIC, Patient Assessment of Chronic Illness Care.

a Summary scores were calculated after multiple item-level imputation to accommodate missing data.

b Pre-workshop sample size is 435, whereas post-workshop sample size is 455.

c Regression coefficient for site (referent = non-implementation) controlling for covariates. All covariates were centered on the grand mean.

Following process evaluation, we analyzed clinicians’ and patients’ perspectives at the implementation site for insights into why there was no substantial difference in person-centered care between sites. Insights include: (1) clinicians wanted to see kidney symptoms, not QOL; (2) workshops were tailored to clinicians’ educational needs, not patients’ needs; and (3) variable use of ePRO data. Illustrative quotes are provided in Table 5, along with exemplars of negative/alternative cases (when applicable) to represent a diversity of perspectives.

**Table 5.** Illustrative quotes by insight.

Sub-topic	Illustrative quote
Insight 1: Clinicians wanted to see kidney symptoms, not quality of life	
Symptom focused	“Like holistically, for me, it would be really good to know if somebody’s in chronic pain. You know, and linking that to depression and anxiety, or is not sleeping. That would be helpful to know.” (Clinician 4, Clinician Focus Group 1, q1)
	“So the only part that you guys, as nurses, are able to control is that dialysis part of it. I mean, like there’s a relationship and everything like that, and just having a person feel like they’re being cared for can improve that as well. But if we’re actually not able to take any action on some of these things – Why ask? Then we’re falling short, right?” (Clinician 5, Clinician Focus Group 1, q2)
	“Like all the uremic symptoms and stuff like that, are already been assessed. And that’s the part of the clinical assessment that we have to ask them. . ..I think weight gain, in-between dialysis runs. Blood pressure. . .more about hypertension. What else? Just like what they said, about GI [gastrointestinal] as well.” (Clinician 4, Clinician Focus Group 2, q3)
	“Quality of life – like it’s just vague. Like, I’m wondering if it should even be in this overall grid [ePRO]. ‘Cause it’s just – it doesn’t – it’s not a symptom. And it’s not – like something that’s gonna bother you.” (Clinician 1, Clinician Focus Group 1, q4)
	Negative or alternative cases: “There should be a general, ‘How are you doing?’ Which this [ePRO] does kind of address, but only if they – address it! . . . Overall, this is mostly fine. You could add things to it all day, probably . . . But – they need to be aware of it, and at least show us that they’re aware of it.” (Patient 1, Patient Focus Group 1, q5)
Presentation of ePRO data	“If you could somehow graph the results, that would be more useful.” (Clinician 4, Clinician Focus Group 1, q6)
	“But being able to go back on yourself and look months prior or something I think, would be very beneficial to track our situation, you know? ‘That hasn’t changed for three or four months, and maybe I could speak with them about that and see what we can –’ you know?” (Patient 3, Patient Focus Group 1, q7)
Insight 2: Workshops were tailored to clinicians’ educational needs, not patients	
Workshop 1: ePRO use and integration into clinical practice	
Workshop objectives (Obj), guided by Bloom’s Taxonomy (Knowledge, Skill, Attitude): Define patient-reported outcomes and describe use of ePRO reports in practice (Obj 1.1 – Knowledge)	“I think the key feature is that the – they actually have some value. Um, and that it’s not just constant repetition. If they’re acknowledged by the staff, they actually have some purpose for them as well, I think that could be useful” (Patient 16, q8)
	“I think a lot of nurses were just told, like ‘you have to do this.’ But even they didn’t quite get why.” (Clinician 2, Clinician Focus Group 2, q9)
	Negative or alternative cases: “At the end of the day, if it’s not helping me do my job, I don’t care. [Laughing] And it’s pretty low on my list. If you tell me I have to do it a certain amount of times, then I’ll probably do it. . .If it can be presented to staff in a way that they really understand that this gonna help them get to the bottom of what we’re trying to do, then I think people will just –” (Clinician 1, Clinician Focus Group 2, q10)
Demonstrate inviting a patient to complete an ePRO, as well as responding to ePRO information (Obj 1.2 – Skill)	“I find that this, ‘What’s your well-being?’ most of them, they, they just – they have great difficulty understanding that general concept. And I think, partly for us too, it’s – it’s hard to explain it in something concrete for them to understand.” (Clinician 4, Clinician Focus Group 1, q11)
	“I’d [like a] tip on how to explain this [ePRO] to patients, to explain the wording so that you get a true picture.” (Clinician 4, Clinician Focus Group 1, q12)
	“For myself, like I said, they’ve [clinicians] never referred to this. And my answers have varied . . . The answers have been different. And there’s been no – I have to bring up the issues if I want to talk about them.” (Patient 4, Patient Focus Group 1, q13)
	Negative or alternative cases: “I find people are much more honest when you leave the room. So, in the few – previously to this ah, study, I always gave them the opportunity to fill it out when I wasn’t in the room, but looked at it before the physician arrived.” (Clinician 2051, q14)
Explain to a patient how ePROs are used in your practice (Obj 1.3 – Attitude)	“As opposed to simply just giving it to [patients], I would suggest that it be noted: ‘This is something we do on a regular basis. We’d like you to fill it out. And we’d like to talk to you about it afterwards,’ which I think is a bit of fair warning. Because when you’ve never talked about it before, it’s like, “Oh, we’re actually gonna discuss this. Oh, okay, well maybe that will give me a different perspective on what I’m filling out.” (Patient 16, q15)
	“I wonder how much the patient understands. Because we ask the same questions [during clinical assessment] and they give us, it’s – it contradicts what they filled out [in ePRO].” (Clinician 3, Clinician Focus Group 3, q16)
	Negative or alternative cases: “I had actually spent an entire morning one day preparing a version of [ePROs] from NIS [Nephrology Information System] to show a patient that PD wasn’t working for him, and had never worked for him. And the visual of being able to show it to him really opened his eyes. And he’s actually switched to hemo and he’s much happier. Having this [trend report] for me that day in particular, would have been exceptionally handy, just to be able to show him the trends. Um, ‘cause it was like, like, 14 months I think, worth of data that I showed him that day. And so, pulling all of that from NIS [Nephrology Information System], printing it, scaling it – it took quite some time. But it was very valuable.” (Clinician 2, Clinician Focus Group 5, q17)
Workshop 2: Patients’ valuing of, and relationship to, use of ePROs in their care	
Justify the value of ePROs use for patients themselves, and in shared decision-making (patient with practitioners) (Obj 2.1 – Knowledge)	“It’d be interesting to know patients’ perspective on this whole thing and what do they want us to be doing with this information. That can help us inform our own practices.” (Clinician 1, Clinician Focus Group 5, q18)
	“Like I just figured they were using it as a survey.” (Patient 1, Patient Focus Group 3, q19)
	“I’m not even sure what difference these questionnaires make.” (Patient 1, Patient Focus Group 2, q20)
	“Somebody’s actually looking at it, yeah. And thinking about it, like you say. It would make me feel a little more like they care . . .. Well, not only that, if you’re – ‘Oh, they do care. They do look at all that. We’re not filling that out just for –’ ” (Patient1, Patient Focus Group 1, q21)
	In answering any of the [ePRO] questions, I’m giving an idea where I feel I am, in terms of living. Um . . . to see how my overall treatments are going.” (Patient 25, q22)
Navigate course of action / follow-up with patient when significant symptoms identified in ePRO (Obj 2.2 – Skill)	“They [clinicians] weren’t clear, and they said ‘Oh it’s like, like –’ like they explain that is was for a greater research program but they didn’t really understand the context of it any better than I do.” (Patient 2, Patient Focus Group 3, q23)
	“There’s an expectation I think, [by] a patient that, ‘Oh, I’m being asked about it, so there’s interest there. So then, there’s some follow-up –’ Maybe there’s the expectation that we can actually attend to some things when we’re not equipped to actually attend to it.” (Clinician 7, Clinician Focus Group 3, q24)
	Negative or alternative cases: “After the assessment and we get this [print out], then we will have the time to ask the patient. ‘Okay, so you told me about the assessment, that this is, ah, what you have rated yourself. And then, with this ah, assessment, you put your number’– let’s say, eight – “Can you tell me more about this? Like, is there anything that you didn’t understand? Or is there anything that you didn’t tell me?” (Clinician 2, Clinician Focus Group 3, q25)
Align practice with belief or value that care encompasses the entirety of a person’s needs and preferences, beyond medical or clinical dialysis care (Obj 2.3 – Attitude)	“I was expressly told, ‘you don’t come and talk to us about things that aren’t related to kidneys. Because everything that you’re experiencing isn’t related to your kidneys. So, don’t talk to us about arthritis, don’t talk to us about hypertension, don’t talk to us about pain issues.’ ‘Kay, but I can’t talk to my GP about them because they’re worried they’re going to screw up the kidney. [Laughing] So, it’s got to be integrated. Because trying to work under these things and vacuums just doesn’t work.” (Patient 2, Patient Focus Group 4, q26)
	“So that’s my point – is, if we had training and tools. So I – because I’m hearing a bunch of different things. One is there are issues that maybe we should ignore because we can’t deal with it properly. [laughing] Or that we shouldn’t, or we shouldn’t address them because we don’t have the skills to do so. But those could also be opportunities. (Clinician 6, Clinician Focus Group 4, q27)
	“Like for example it says ‘Worst possible depression.’ Sometimes I put ‘10.’ And I got no response from anybody.” (Patient 4, Patient Focus Group 4, q28)
	“I would rather someone said, ‘I really don’t know what to do with that’ than look at you blankly, or ignore it” (Patient 16, q29)
	Negative or alternative cases: “I have a patient right now that had been experiencing pain. But I don’t think it’s actually related to dialysis. So what I do, instead of like refer this patient to the nephrologist. . .I told this patient, ‘Okay, you go and visit your family doctor and get a proper referral to so-and-so. Because I already know that it’s not related to dialysis.” (Clinician 2, Clinician Focus Group 3, q30)
Workshop 3: Strategies of ePROs to support communication or coordination of care	
Plan use of ePROs to support communication within and beyond the home dialysis team (Obj 3.1 – Knowledge)	“It’s very nurse-heavy here. So, I’d be interested to hear, does the social worker use it? Does the dietician use it? If the nephrologist or resident of nephrology have access to it on NIS [Nephrology Information System], would they utilize it? What would they do with it? Um, or are they just slowly relying on us to do it I think, ‘cause we always have, maybe? I don’t know’.” (Clinician 2051, q31)
	“I’ve seen it at clinics before . . . being used. But I’ve never thought – like, it’s something that we’re recording. I thought it was just something that the nurses will use, just for them to have. Like I didn’t know it was something that should be recorded at some point that we [Nephrologists] should be following up on.” (Clinician 7, Clinician Focus Group 4, q32)
	Negative or alternative cases: “You could spend hours there discussing different routes. But ah, you know, by doing the questionnaires, at least she [clinician] knew which areas to pinpoint” (Patient 25, q33)
Practice assessment and follow-up when multiple challenges are identified in an ePRO (Obj 3.2 – Skill)	“If I live with this all the time, I don’t know that I should report it? And, you know, ah – in a weird way, what do the doctors wanna know? Because I think there’s a disconnect there as well.” (Patient 16, q34)
	“Most nephrologists have chosen to go into a career such as nephrology as a specialty, as opposed to primary care. And so, these things cover things that are well outside of the purview of what most nephrologists feel comfortable dealing with. So, you know, nausea, drowsiness, tiredness may relate very much to uremia and to something that would definitely fall under our umbrella. But pain doesn’t. And pain isn’t something that most of us have a desire to deal with, or are equipped to deal with. And so, there’s actually a fair bit of push back to people cramming pain down our throats. So, I think that’s an important element. You know, things such as anxiety, depression – you know, best well-being – those aren’t things that we’re particularly well adept at managing. So, the truth is, most nephrologists, my guess is, would just as soon not hear about things that don’t really fall into their scope of practice.” (Clinician 4, Clinician Focus Group 3, q35)
Integrate an awareness of ePRO use within one’s own practice to support communication within and beyond home dialysis team (Obj 3.3 – Attitude)	“If there was a means to attach a trend report to a case summary, like a visual that they could send to the GP, that would be fantastic. I’m not sure if that’s doable with our current systems, but -” (Clinician 4, Clinician Focus Group 5, q36)
	“I don’t see the social worker as a good source of mental support.” (Patient 1) “Yeah. It’s the ah, I was thinking they should refer you to, to speak to somebody in mental health or something, you know.” (Patient 2, Patient Focus Group 1, q37)
	Negative or alternative cases: “So what it means is that we’re given a whole bunch of information, a whole bunch of data. And then we’ve got our hand tied behind our back because we don’t have good links with palliative care. We don’t have good links with psychiatry. We don’t even have good links with orthopedic surgery. We have reasonable links with vascular access, because that is in our primary wheelhouse. But so, it doesn’t necessarily help anyone if we get a bunch of information and then, we’re left stuck saying okay, now duty of care falls on us.” (Clinician 4, Clinician Focus Group 4, q38)
Workshop 4: Routine integration of ePROs is a fundamental change to practice	
Describe how routine integration of ePROs is a fundamental change to practice (Obj 4.1 – Knowledge)	“When it comes down to making the clinic run on time, so the physician can get in to see the patient and we can get to the next patient, what ball can be dropped? Well, this ball can be dropped. It’s not direct nursing care. It’s not – you know, the dressing that no one else can do, it’s not the transfer set change that no one else can do, it’s not the symptom assessment that no one else can do, right?” (Clinician1, Clinician Focus Group 1, q39)
	“And I ask, to be honest with you, because after the 3^rd^ time it’s like, okay, hmm, ‘does it make any difference – how I feel’? And [the clinician] is like, ‘Oh no, we just put it on record and . . .’ ‘Okay, but how does that help me?’ And nobody really answers.” (Patient 3, Focus Group 2, q40)
	Negative or alternative cases: “Yeah, to be very honest with you, I honestly ah, ah yeah, it didn’t occur to me as a relevant tool until it started to resurface that we were missing part in our practice the last few years. I didn’t think it was relevant to evaluate. . .not something I thought was relevant for decision-making.” (Clinician 2, Clinician Focus Group 4, q41)
Integrate ePROs into clinical interview (Obj 4.2 – Skill)	“They were laminate (ePRO on paper) and washable so, they weren’t even permanent, right? So, I don’t know, how long were they permanent for?” [Note: laminated paper was used by the clinic for PRO collection prior to ePRO collection.] (Patient 2, Patient Focus Group 4, q42)
	“I don’t even look at this until I go to enter it [into Nephrology Information System], which might be hours later. Be – there’s – just because of the time management perspective, we’re more focused on the symptomology that we assess as part of that clinic sheet that you were talking about. Because that’s what the doctor cares about, right?” (Clinician 2, Clinician Focus Group 1, q43)
	Negative or alternative cases: “When the patient was complaining about something all the time, I asked what the trend was over a period of time, over the last several visits. At least she told me that this is how it has been . . . This has been consistent over the last say, six months or something.” (Clinician 3, Clinician Focus Group 4, q44).
Prioritize patients’ self-reports as complementary to one’s own clinical assessment (Obj 4.3 – Attitude)	“And I find a lot of times, what [patients] say on this one [ePRO], rating one to ten, and what they say before for the clinic sheet [clinician checklist], contradicts.” (Clinician 3, Clinician Focus Group 1, q45)
	“If this information were fed into, a central file, my file . . . I could see the trends myself. Ah, I mean, I live those trends, but I don’t necessarily remember what – how I was two and a half years ago, or something like that. But if that were fed in so that I – there’d be a running check on my progress, or lack thereof, then I’d – get some indication of whether or not I’m adhering to the protocols or – or not. (Patient 2, Patient Focus Group 2, q46)
	Negative or alternative cases: “Half an hour prior to the nephrologist coming, I’ll use both of those things (ePRO and clinician checklist) to fill out my clinic sheet. And then go, ‘Oh, look. You have a seven out of ten pain scale. Tell me about that.’ So I use it in conjunction with, if I can. But it’s only happened a couple of times, so -” (Clinician 1, Clinician Focus Group 3, q47)
Insight 3: Variable use of ePRO data	
Clinicians’ perspectives of use of ePROs after workshops	Interviewer: “Have you changed the way you’ve used ePROs in the last year?” Clinician: “Quite honestly, no.” (Clinician 2014, q48)
	“We’re doing this because it’s something we are asked to do. . .Let’s say, for example, you’ve rated pain for seven – and I said, ‘Seven? That’s too high for me. Are you on any pain medications?’ ‘No.’ And I said, ‘So what is [an] acceptable number for you?’ And he was like, ‘Five or six.’ So, in other words, his standard at that point is not too high from what he rated his pain symptoms at that point. Then you come to like, ‘Oh, these [ePROs] are pretty useless tools then.” (Clinician 2005, q49)
	Interviewer: “You mentioned that if [the patient] is not in the study, you won’t [invite them to complete PRO] on the laminated paper.” Clinician: “I have mostly been just using the ePRO with the study patients. There’s a few nurses that haven’t used the laminated [PRO on paper] if they [patients] weren’t in the study.” (Clinician 2003, q50)
	“I can’t say [use of ePROs] has changed. Um, I mean it maybe has made me more aware.” (Clinician 2050, q51)
	“Before the study, I would look at [ePROs] after the fact. And then, the patient had gone home. But now – they just made us more aware, maybe just to look at it. And address it.” (Clinician 2013, q52)
	“I think my practice has changed a little bit in the sense of it being less data entry and more patient-focused. . .Knowing that the patient’s perspective can be highly different than what we usually find when the physician or the RN does the checklist and is clinically prompted. Having the patient’s perspective first [due to ePRO completed before appointment] has changed the way that I assess them and the way that I ask questions. I would say that it’s developed a little bit more of a trusting relationship in some sense because I can see them as a whole, and having a bit more impact on their quality of life. (Clinician 2011, q53)
	“I think it’s definitely got value . . . in coordinating care and – finding out, you know, the state of our patient – like, ‘how are they doing?’ Not just, ‘are they walking in okay? Do they look okay? Is their blood pressure okay?’ It kind of looks at them as more as a person, you know, it’s mental health, and social, and physical. It’s more holistic, for sure.’ (Clinician 2029, q54).
Patients’ perspectives of use of ePROs after workshops	“I’m not sure how widely they’re shared. But if my assessments could be or would be shared with my social worker, or my nutritionist, or these other supporting people, um, that are out there, um, I think that that’s helpful as well . . . The more people that have access to the information, ah, I’d be comfortable with that. (Patient 119, q55)
	“And it makes your time feel valued if they’re actually referring to it. In my case, it feels like I understand the potential benefits. But it’s not being referred to, so it feels like I’m wasting my time.” (Patient 5, Patient Focus Group 7, q56)
	“It’s just we have to take it one step further, and get the practitioners, or whatever, to change the way they do business a little bit. And also, give the, sort of the, I hate to say the ammunition here but the, ah, to make it real – also, myself responsible for asking questions.” (Patient 204, q57).
	“The minute I mark whatever number 2 to 10, whatever it is, they should ask me. They should address it . . . .” (Patient 3) “And if they don’t, then you go, ‘Okay. I guess it was more important to me than it probably should have been.’ And you kinda forget about it, right? . . . ” (Patient 2) “If I’m reporting something as a four or five, I kinda expect them to, you know, bring it up, so I can talk about it. . .” (Patient 1) “When you get an answer, you can make an assessment for yourself kind of, how serious it is. So when they don’t address it, you’re kinda left in that kind of limbo, right? Like I’m being maybe more anxious about it than I need to be. . .We’re not always equipped; we’re not doctors. So, we’re not always equipped to know if that is serious, or if that’s just a minor inconvenience. So, when you put it down on the sheet, you’re expecting them to interpret that information, ‘Is this serious? Or is it just something you’ve gotta live with?’ Right? The two opposite extremes.” (Patient 2, Patient Focus Group 8, q58).
	“I’m hoping that they’re going to adopt them and see them as a method of, um, being able to support the patients where they are, so that, um, especially with the ability to see trends and changes, that it will become an automatic part, I should say, it should be a mindful part of the patient and clinician experience.” (Patient 1, Patient Focus Group 8, q59)
	“The penny didn’t drop right away. It did, eventually. And it was like, ‘Oh, I think this really does have some real power to it’ when we recognize that it can be a part of what we do, ah, to take care of ourselves.” (Patient 16, q60)
	“To be honest, doing [ePROs] ahead of time is actually degrading the opportunity for us to give them feedback. Because the old, when we would mark on the erasable board, well, it’s right there in front of us. It’s an opportunity for us to actually interact with them. This way, they get this. They never have the opportunity to talk. They see it in advance. Never shows up during the visit.” (Patient 3, Patient Focus Group 9, q61)

ePRO, electronic patient-reported outcome; GP, General Practitioner; PD, peritoneal dialysis; RN, Registered Nurse.

#### Clinicians wanted to see kidney symptoms, not QOL

For 8 years prior to the study, the implementation site had collected the ESAS-r:Renal on laminated paper during appointments, and the KDQOL-36 annually. Both were entered into an electronic health record; clinicians did not see KDQOL-36 responses, but sporadically saw the ESAS-r:Renal. During initial focus groups, clinicians discussed what additional health or QOL information they might like patients to provide. Clinicians chose to see the questionnaire they were most familiar with, the ESAS-r:Renal, not the KDQOL-36 or EQ-5D-5L. The ESAS-r:Renal items address 11 symptoms, plus one on wellbeing. Clinicians requested usual clinic questionnaires [ESAS-r:Renal, visual analog scale (0–100) from EQ-5D-5L, and four questions about activities of daily living (bathing, dressing, walking, transferring bed to chair)] be completed simultaneously online to aid workflow. Kidney symptoms were the priority, perceived as something clinicians could ‘control’ [quote(q)1–3], whereas QOL was ‘vague’ (q4). Patients were asked what other information would be important. They emphasized their desire for a relational connection, to be asked ‘How are you doing?’ They wanted clinicians to acknowledge the ePRO responses they were already providing (q5).

Prior to each appointment, patients completed the ESAS-r:Renal. Although clinicians had online access via Cambian, they asked for ePROs to be printed. They did not have computers in all the clinic rooms, shared computers in the hemodialysis clinic, and used a hybrid paper/electronic chart. Thus, the ESAS-r:Renal was printed and handed to the nurses. Nurses entered the ESAS-r:Renal responses into electronic charts after appointments. Clinicians later asked to see ESAS-r:Renal trends over time, printed in color (q6). All patients had online access to their data, and many commented on the benefits of seeing their responses over time (q7).

#### Workshops were tailored to clinicians’ educational needs, not patients’ needs

Workshops were tailored to multi-disciplinary clinicians’ educational needs. Workshop objectives were guided by Bloom’s taxonomy to address knowledge, skills, and attitudes,^45^ further informed by extant literature on clinician training to use PROs, and through consultation with team experts. (See Table 5, column 1, for workshop objectives.) Workshop locations, format, length, timing (for shifts), frequency, learner-activities, and door-prize-incentives were co-created with clinicians.

Workshop 1 addressed ePRO use and integration into practice. Patients and clinicians alike did not understand that after completion, clinicians should acknowledge, review, and discuss responses with patients (q8–10). Neither group understood why patients were completing ePROs; clinicians did not know how to invite patients to complete ePROs nor that patients should answer them by themselves (q11–14). An invitation script, informed by the data (q15), was provided and clinicians said a personalized version to each other. Attendees reviewed strategies to use patients’ responses to inform patient-clinician interactions (q16–17).

Patients co-presented Workshop 2 focusing on patients’ valuing of, and relationship to, use of ePROs in their care. Patients read quotes about actual and envisioned use in shared decision-making (q18–22). Attendees discussed case scenarios when ePROs identified significant symptom concerns (q23–25), especially when clinicians felt ‘not-equipped to actually attend to it’ (q24). ePROs identified needs outside of traditional dialysis care, such as pain, depression, anxiety; clinicians debated whether they may attend to the entirety of a patient’s needs (q26–30).

Strategies were discussed in Workshop 3 for use of ePROs to support within-team communication or coordination of care. While nurses typically looked at ePROs first, attendees discussed how ePROs could support communication within home dialysis (q31–33). Clinicians brainstormed follow-up when numerous challenges were identified for one patient through longitudinal ePRO responses (q34), and what fell outside scope of dialysis care (q26, 27, and 35). Clinicians considered and challenged ePRO use to support communication beyond the home dialysis team (q35–38).

Routine integration of ePROs as a fundamental change to practice was discussed in Workshop 4. Given ePROs were often not seen as important or useful (q39–41), attendees discussed whether this information could provide a ‘missing part in our practice’ (q41), and optimal ways to integrate ePROs into workflow (q39, 42–44). ‘Contradictions’ between ePROs and clinicians’ assessments were debated alongside strategies to use ePROs as complementary to clinicians’ assessments (q45–47).

#### Variable use of ePRO data

**Figure 4.**
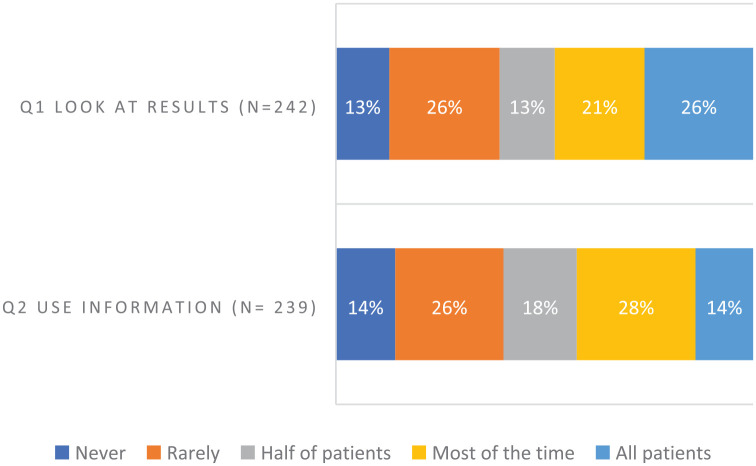
Clinicians’ reports of viewing and using ePRO data. In phase II, clinicians at the implementation site were asked to anonymously answer the below rating-scale questions. They were invited every 2 weeks to answer. Responses were anonymous; *n* = 244 denotes the number of submitted responses (215 responses from allied health care providers; 29 responses from nephrologists); Q1 had two missing; Q2 had five missing). Questionnaire items: 1. In general, how often over the past 2 weeks did you look at the PRO results? 2. In general, how often over the past 2 weeks did you use the PRO information to inform or guide the care you provided to patients? ePRO, electronic patient-reported outcome.

Despite tailored educational support, use of ePROs was variable. In phase II, clinicians were invited every 2 weeks to anonymously answer two rating-scale questions. They did so a total of 244 times; 47% of the time they looked at ePRO results most or all the time, and 42% of the time they used ePRO information most or all the time (Figure 4). In summary, ePRO data were viewed or used less than half of the time.

**Figure 5.**
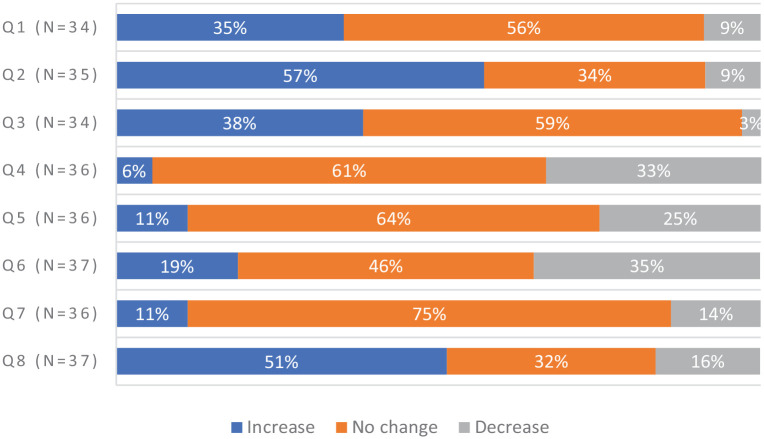
Change in clinicians’ knowledge and beliefs about use of ePROs. Clinicians who attended workshops were invited to complete a questionnaire regarding their knowledge and beliefs about use of ePROs. The first questionnaire was completed before they attended a workshop, and the second questionnaire was completed after all workshops were delivered. % increase = the increase % of respondents who provided an affirmative response to any of the questionnaire items. Questionnaire items: 1. Do you look at these PROs? 2. If you were asked to explain to a home dialysis patient why they were completing the patient-reported outcomes, and what was going to be done with their reports, how would you assess your own skills? 3. Today, how competent would you feel if you were asked to follow up with a home dialysis patient regarding their responses in their patient-reported outcomes? 4. It is very important for home dialysis programs and practitioners to use patient-reported outcomes. 5. Person-centered care in home dialysis can be enhanced by routine integration of patient-reported outcomes. 6. It is my responsibility to review and respond to a patient’s quality of life concerns beyond those traditionally connected to kidney disease (i.e. pain, depression, anxiety). 7. It is reasonable to believe that a patient’s reports of their health, quality of life, and experiences of care may change over time, from one clinic appointment to the next. 8. A patient’s report of their symptoms, quality of life, and experiences of care would be reported exactly the same as if a healthcare practitioner reported on their behalf (i.e. in proxy). ePRO, electronic patient-reported outcome.

Clinicians’ knowledge and beliefs about use of ePROs, compared before and after workshops, showed disparate uptake: 35% of clinicians reported an increase in looking at ePROs, 57% improvement in skills explaining ePRO completion to a patient, 38% greater competence in follow-up, and 51% greater understanding that patients’ self-reports differed from proxy reports. However, 33% found less importance for ePROs being used by kidney programs or practitioners, 25% less enhancement of person-centered care with routine ePRO use (64% no change), and a 35% decrease in clinician responsibility to respond to ePROs (Figure 5).

After the workshops, clinicians’ narratives about how they used ePROs was clinician dependent, spanning the spectrum of person-centered kidney care. On one end, clinicians did not change how they used ePROs (q48), did not consider them useful (q49), and no longer invited patients to complete ePROs if they were not participating in the study (q50). The majority of clinicians expressed increased awareness (q51), and looked at responses prior to a patient leaving the clinic to ‘address it’ (q52). On the other end of the spectrum, some felt their practice had changed and that ePROs facilitated their engagement with a person ‘as a whole’ (q53 and 54).

Patients did not know who saw their ePRO responses (q55), which were frequently not acknowledged (q56). Patients were left wondering if they could or should bring them up, or if that was the clinicians’ responsibility (q57 and 58). Patients saw value in completing ePROs, appreciated seeing their trends over time, and envisioned ePROs supporting ‘a mindful part of the patient and clinician experience’ (q59) or self-care (q61 and 62).

## Discussion

We found that person-centered care was not enhanced through educational support for multidisciplinary kidney clinicians to routinely use ePROs. Overall PACIC score and domain scores were similar at implementation and non-implementation sites throughout the study, including after clinician workshops. While the extant PRO literature emphasizes training clinicians,^2,19–23^ our findings show that this alone seems insufficient to show enhancement in person-centered care. The qualitative findings confirm the PACIC analysis, and offer explanatory insights for this departure; each of the three insights are discussed in relation to literature in the field.

First, clinicians wanted self-reports on kidney symptoms, not QOL. Similar to many chronic illnesses, treatment of symptoms and use of technology has reified ‘dialysis-centered care’.^46^ Health systems reinforce this by care being organized by body parts; clinicians specialize accordingly. Understandably, clinicians wanted to see what they were familiar with. They may also have wanted to see which kidney symptoms lowered QOL, so they could be addressed (if within perceived scope of practice). Reports about non-dialysis concerns (i.e. pain, depression, anxiety) left clinicians feeling ill-equipped, a finding previously reported.^1^ In a similar study on use of ePROs in home dialysis in Canada, nurses also chose to look at symptoms (ESAS-r:Renal) over QOL (KDQOL-36) even when both were provided.^5,6^ While clinicians were symptom-focused, patients wanted their responses to be acknowledged and personalized. They wanted to be asked ‘How are you?’ and really listened to. Improving patient–clinician communication was also identified as a top challenge in in-center hemodialysis in Canada.^47^ But dialysis patients’ perspectives on use of PROs to facilitate communication or in their care has rarely been explored.^1,4,8^ Clinicians’ focus on symptoms, regardless of wider training of routine integration of ePROs into care, may have truncated movement toward person-centered care.

Second, workshops were tailored to clinicians’, not patients’, educational needs. While patients’ perspectives and the extant literature informed this design, clinician’s knowledge, skills, and attitudes were targeted. While clinicians are a part of person-centered care, they are one part, not the whole. A focus on person-centered kidney care necessitates a culture shift from a clinician or system orientation, toward shared emphasis with patients’ values and priorities.^48^ But this is a fundamental change to kidney practice, re-orienting delivery from symptom-centered or dialysis-centered to person-centered.^9,49–51^ Future patient-oriented research may focus on empowering the person receiving dialysis to routinely use ePROs, an approach not previously explored.

Third, clinicians’ use of ePRO data was variable, only looking at and using it less than half of the time. To address ongoing questions about use of ePROs in routine kidney care, we created two animated videos, one for clinicians and one for patients, which were made available after the study was completed (https://www.healthyqol.com/kidney). In the global movement toward person-centered kidney care,^1,48–51^ use of PROs by clinicians may ultimately be less important than addressing systemic challenges in dialysis care that hinder person-centered practices, such as traditional provision of healthcare where clinicians’ knowledge or biomedical information is privileged over patient knowledge, and healthcare organized in silos according to body parts (i.e. kidney, cardiac, mental health).^26^

The strengths of our study include longitudinal ePRO collection in a large sample alongside a mixed methods approach for data triangulation with insights illuminated through strategic approaches to rigor, further informed by the Knowledge-to-Action Framework,^30^ including credibility (i.e. iterative, co-development of an educational support with clinicians, use of multiple data sources, attention to negative case analysis); confirmability (i.e. reflexive field notes, audit trails during data collection and analysis, interviews with research assistants who collected data at the two sites); and transferability (tailoring workshops to local context while leveraging extant literature). Nevertheless, our study is not without weaknesses. Although clinicians received training on integration of ePROs into care, they elected to only receive an ePRO focused on symptoms. In addition, clinicians at the implementation site had previously collected this information for 8 years prior to the study, and it is unknown whether this impacted findings. An ePRO focused on symptoms may not have been the optimal information for clinicians to see, and it may have limited a person-centered approach to kidney care, which is intended to encompass the whole of a person.^9–13^ The PACIC-20 showed that care was already very good in both sites, thus improvement may have been untenable. Furthermore, the lack of integration of the ePRO digital health platform with the electronic medical record system used by the clinicians at the implementation site may have impacted their receptiveness to routine use of ePROs. Finally, this study was conducted prior to the COVID pandemic. With the wide-scale adoption of telehealth options during the pandemic, future studies may investigate whether in/acceptability of telehealth innovations in kidney care impacts routine use of ePROs.

## Conclusion

The results of this study reveal that training clinicians on the use of PROs is complex and likely only part of what is required to enhance person-centered care. While this is counter-culture to the extant literature that encourages clinician training for routine collection, use, and integration of ePROs, person-centered kidney care may necessitate a wider strategy that addresses the complexity of such a culture shift. Our findings provide an important contribution to the literature because we identified three insights to explain why this might have occurred. First, clinicians wanted self-reports on symptoms, not QOL. Second, workshops were tailored to clinicians’ educational needs, not patients. Third, use of ePRO data by clinicians was variable. Our findings emphasize the need for research that emphasizes patients’ priorities in using their self-report to inform their care. While ePRO use has the potential to improve patient outcomes, it seems to require intervention beyond clinician education.

## Supplemental Material

sj-docx-1-taj-10.1177_20406223231173624 – Supplemental material for Electronic patient-reported outcomes in clinical kidney practice (ePRO Kidney): a process evaluation of educational support for clinicians Click here for additional data file. Supplemental material, sj-docx-1-taj-10.1177_20406223231173624 for Electronic patient-reported outcomes in clinical kidney practice (ePRO Kidney): a process evaluation of educational support for clinicians by Kara Schick-Makaroff, Scott Klarenbach, Jae-Yung Kwon, S. Robin Cohen, Joanna Czupryn, Loretta Lee, Robert Pauly, Jennifer M. MacRae, Bruce Forde and Richard Sawatzky in Therapeutic Advances in Chronic Disease

## Declarations

**Ethics approval and consent to participate:** Ethical approval was granted by University of Alberta / Alberta Health Services (#Pro00068365) and the University of Calgary (#REB17-0506). Informed written consent was obtained at the first point of data collection and re-confirmed at subsequent interactions.

**Consent for publication:** Not applicable.

**Availability of data and materials:** Ethical approval was not attained for the purposes of public sharing of the qualitative data. Upon completion of analysis, anonymous, quantitative data will be made available online indefinitely to the public on a database. Scientists who are interested in using the data may contact the corresponding author for further information.
