# Supplementary material for: Electronic patient-reported outcomes in clinical kidney practice (ePRO Kidney): a process evaluation of educational support for clinicians
Source: Ther Adv Chronic Dis. 2023 Jun 12;14:20406223231173624. doi: 10.1177/20406223231173624 (PMC10272664; doi:10.1177/20406223231173624)
Supplement: sj-docx-1-taj-10.1177_20406223231173624 – Supplemental material for Electronic patient-reported outcomes in clinical kidney practice (ePRO Kidney): a process evaluation of educational support for clinicians [file sj-docx-1-taj-10.1177_20406223231173624.docx]

**Electronic patient-reported outcomes in clinical kidney practice (ePRO Kidney):**

**A process evaluation of educational support for clinicians**

**Supplemental Materials**

Contents

[Supplementary File 1. Qualitative Interview & Focus Group Guides 2](#_Toc122503770)

[Phase 1: 1^st^ Clinician Focus Group 2](#_Toc122503771)

[Phase 1: 2^nd^ Clinician Focus Group 2](#_Toc122503772)

[Phase 1: 3^rd^ Clinician Focus Group 3](#_Toc122503773)

[Phase 1: 4^th^ Clinician Focus Group 3](#_Toc122503774)

[Phase 1: Nephrologist Focus Group 4](#_Toc122503775)

[Phase 1: Patient Focus Group / Interview 4](#_Toc122503776)

[Phase 2: Clinician Interviews (for those who attended workshops) 5](#_Toc122503777)

[Phase 2: Clinician Interviews (for those who did not attended workshops) 6](#_Toc122503778)

[Phase 2: Patient Focus Group / Interview 6](#_Toc122503779)

[Supplementary File 2. COREQ (Consolidated criteria for reporting qualitative research) 32-item checklist 7](#_Toc122503780)

# Supplementary File 1. Qualitative Interview & Focus Group Guides

The interview and focus group guides were semi-structured and potential prompts are noted below.

“Questionnaires” were defined as quality of life assessments in the introduction to the interview or focus group.

## Phase 1: 1^st^ Clinician Focus Group

1. I understand that your current practice is to have patients complete the questionnaire before each clinic visit.

- How is this done (the process)?
- Is this questionnaire used with the patient during their clinic visit? How?
- What is your understanding of why you collect this information from patients?
- Are there times when it is not consistently used? What do you do?
- What are your thoughts/feelings on how this patient information is being collected and used?

1. When the questionnaire was first introduced, did you receive any training?

- If so, did you find it helpful?
- Were there any aspects of the training that were missing or didn’t help?
- If there was no training, what would have supported you in incorporating this questionnaire into your daily workflow?

1. What additional information would you like to receive from patients?
2. Is there anything else that you would like to share about the way you currently use the questionnaire in your current clinic practice?

## Phase 1: 2^nd^ Clinician Focus Group

1. At this time, patients who participate in the study complete the questionnaire before their clinic visit. How is this working?

- Has the way you collect and review the questionnaire changed since the study started?
- Do you notice a difference in your clinic workflow when you receive the questionnaire before the clinic visit?
- How has our data collection affected your clinic current workflow? Do you have any suggestions on how this can be improved?

1. At this time, how are you using the questionnaire information patients provide to you?

- Has your use of the information changed since the study started?
- Do you have time to review responses prior to clinic visit?
- Who reads/reviews this information?
- What happens if questionnaire responses are not given prior to clinic visit?
- Do you have any other suggestions about how you would like the questionnaire to be used?

1. Please look at this print out of a patient’s questionnaire responses (from Cambian). What do you like or dislike? How would you like to see this information displayed?

- Mapping of trends over time?

1. What information from patients about their health or quality of life would like to receive that you are not currently asking them? (Refer to questionnaire).
   - i.e. bowel issues in peritoneal dialysis; transplant status
2. Now that you have shared with us some of the needs and potential gaps in how this questionnaire information is collected and used, as well as potential uses in your daily practice, what type of information or training would be most helpful to you moving forward?
3. Is there anything else you would like us to know?

## Phase 1: 3^rd^ Clinician Focus Group

1. Over the past 6 months, you have been receiving questionnaires from patients who are participating in this study. Can you tell me how this process has been for you?

- How have you been incorporating the questionnaire into your clinic workflow?
- Have you seen any changes in this process over the last 6 months?
- Are there any ongoing gaps in this process that you would like to address?

1. Over the past 6 months, how have you been using the questionnaire within your daily practice?

- Are you finding time to review the individual and trend reports?
- Are you able to interpret the reports? Identify areas of needs?
- Are you able to communicate any concerns from the questionnaire responses to the patient and initiate a discussion?
- How are you communicating any flagged items/trends to other kidney clinicians?

1. Now I would like to take some time to go over those areas of educational need that you may have shared in previous focus groups, as well as those areas of need that have been identified in the literature on patient-reported outcome (PRO) questionnaire collection and use. I will briefly address each area, one by one, and ask you your thoughts on whether you would like to see this item included in a future educational support and in what way. “Workshops” were the form of education most supported.

Do you have any questions?

- (Provide handout with initial draft of workshop content)

1. Do you have any additional comments about your experiences with collecting and using the questionnaire that you would like to share with us?

## Phase 1: 4^th^ Clinician Focus Group

1. We want to track your use of questionnaire information
   - For each study participant’s clinic visit we print out and give you their questionnaire from today and their and trend report from our Cambian system. Currently, you put the patient’s responses from the questionnaire into Nephrology Information System (NIS), and then discard it.
     - Over the next year we also want to collect the following anonymous information:
       - Are you a physician or an allied health member?
       - Did you review the questionnaire and consider them in your care planning?
     - Would you be able/willing to provide this additional information? If so, how often would you be willing to provide it (i.e. each patient, each week etc.)? How would you like to provide it? (i.e. complete it on the report, as a separate document, in your prep chart etc.)
2. Review workshop sessions/materials with clinicians (4 workshops)
   - Brief review of Bloom’s taxonomy on learning (knowledge, skills and attitudes)
   - For each of the 4 workshop plans:
     - What key messages/learning objective(s) stand out for you?
     - What might you like to see adapted?
     - Is there anything missing that you would like to see addressed?
     - Learning activities: do these activities support how you learn best?
3. In the past, what has motivated you to attend a workshop? What would motivate clinicians, such as yourself, to attend a 45 min. workshop?
   - i.e. Food, certificate of attendance, accreditation, handouts/resources

## Phase 1: Nephrologist Focus Group

1. In the past, home dialysis patients were asked to complete a questionnaire as part of their regularly scheduled clinics. What was your experience of using the questionnaire responses, prior to our study?

- Did you have an opportunity to review the patient’s responses prior to seeing them?
- If not, did the nurse verbally review the patient’s responses with you?
- How often were you informed of or did you personally review these questionnaire responses prior to seeing the patient?
- Did you find the questionnaire responses helpful/unhelpful in assessing or treating your patient? In what way?

1. Once our study began, we asked patient participants to complete the questionnaire electronically, and prior to their clinic visit with the intention of providing the patient’s responses to all clinicians, before the patient encounter. Since that time, have you noticed any change in how or when you receive and use this information?

- How consistently are you receiving this information for patients who are participating in the study?
- How satisfied are you with the way you receive this information? (i.e. verbally, reviewing the questionnaire and/or trend reports)
- Have you changed the way you assess and treat your patients, because you had access to this information?

1. Here is the questionnaire (provide paper copies to attendees). But based on previous focus group responses, we are additionally providing trend reports. We can only provide those if they are completed prior to the appointment and if completed on 2 or more separate occasions.

- What are your thoughts on the way the information is presented in these reports? i.e. Is it easy to read and/or interpret the data?
- Do you find these reports helpful in assessing/treating the patient? In what way?
- If unhelpful, do you have any suggestions on how these reports can be improved?

1. Now that you have shared with us some of the needs and potential gaps in how the questionnaire is collected and used in your daily practice, what type of information or training would be most helpful, moving forward?
2. Is there anything else you would like us to know about collecting and using the questionnaire in order to incorporate the patient perspective into your daily practice?

## Phase 1: Patient Focus Group / Interview

1. To begin, can you please describe what happens during your regularly scheduled home dialysis clinic visits?
2. In the past, during your clinic visit you were asked to complete a questionnaire where you provided information about your symptoms and quality of life. (Show paper copies of questionnaire.) What was your experience of completing this questionnaire?

- How did you provide this information? (i.e. by yourself, with the help of your dialysis nurse?)
- When were you asked to complete the questionnaires? (i.e. at the beginning or end of your visit?)
- How satisfied were you with the way you provided this information?

1. How was this information used in your clinic visits?

- Who brought it up? You or clinician?
- Who discussed your questionnaire responses with you?

Now that we have begun our study, you have been asked to complete the questionnaire, along with three other questionnaires, prior to your clinic visit. The first questionnaire (show on paper copy) will be seen by your clinicians, while the other three questionnaires will be seen by the research team only.

1. What is your experience of completing the questionnaires in this way?
2. Now that your clinicians will see your questionnaire responses prior to your visit, would you like your health care team to use your information any differently? In what ways?
3. Who from your health care team should have access to/look at this information?

- Is there anyone else who should have access to this information? (i.e. transplant team, family doctor)

1. How would you like your family/caregiver’s perspectives to be addressed and incorporated in your care?
2. Looking at the questionnaire, what other information would be very important for you to provide to your healthcare team that is not already asked?
3. Looking at the questionnaire, are there questions here that are not important for you or that you do not think needs to be asked or shared with your healthcare team?
4. What would you like to tell staff about how you would like them to use this information that you provide?
5. Is there anything else you would like to share about the information you are providing and about how it can be used to support your ongoing care?

## Phase 2: Clinician Interviews (for those who attended workshops)

1. To begin, can you please describe how you use questionnaire responses in your regular home dialysis practice? (Can you give an example?)

- How has this changed over the past year?
  - How does receiving patients’ self-reports influence your clinical practice?

1. Are there any additional supports that would help you regularly use questionnaire responses as part of your clinical kidney practice?
2. Now, can you please share your experience with attending the clinician workshops?

- Did anything from these workshops stand out for you? If so, please explain/give an example.
- What did you hope to learn or gain from attending these workshops?
- Were there any gaps between what you expected or needed to learn from these workshops and what was presented? If so, please explain/given an example.
- Any suggestions for how the workshops could be changed in the future?

1. How has attending these workshops, or being a part of this study, influenced your perspective on regularly using quality of life assessments in your clinical practice?

- After attending these workshops, are there any ongoing barriers to using this information? If so, please explain/give an example.

1. Is there anything else you would like to share about participating in this study?

## Phase 2: Clinician Interviews (for those who did not attended workshops)

1. To begin, can you please describe how you use questionnaire responses your regular home dialysis practice? (Can you give an example?)

- Has this changed over the past year?
  - How does receiving patients’ self-reports influence your clinical practice?

1. Are there any additional supports that would help you regularly use questionnaire responses as part of your clinical kidney practice?
2. As a part of our study, we offered workshops for clinicians to learn about and discuss use of the questionnaire in kidney practice. Were you aware of these workshops?

- If yes, can you share with me either why you choose not to attend, why you were unable to attend, or what we could change in the future to support your participation in the future?
- If no, would you like to receive information directly from us regarding future workshops?

1. How has being a part of this study influenced your perspective on regularly using questionnaire responses in your clinical practice?

- Are there any ongoing barriers to using this information? If so, please explain/give an example.

1. How would you like the questionnaire to continue to be used in home dialysis, after the study is complete?
2. Is there anything else you would like to share about participating in this study?

## Phase 2: Patient Focus Group / Interview

- - - 1. To begin, how do your home dialysis health care providers respond to your questionnaire responses?
- Over the last year, have you noticed any changes in how your questionnaire responses are used in your care?
  - - 1. When you saw your health care providers responding to your questionnaire responses (or perhaps not responding), what was *your* response?
      2. What advice would you give your home dialysis health care providers on how they could use your questionnaire responses to meet your specific health needs?

1. How do you use your questionnaire responses in your interactions with your home dialysis health care providers?

- Can you share an example?
- Has your completion of the questionnaire changed the ways in which you care for yourself? If yes, in what way?

1. How would you like your home dialysis health care providers to respond to quality of life concerns perhaps not typically associated with kidney disease or dialysis, such as pain, anxiety or depression?
2. Based on your experiences completing the questionnaires as part of our study, are there any supports you would like to have in place, either for yourself or your healthcare provider, for use of your responses?
3. How would you like your home dialysis health care providers to continue using your questionnaire responses after our study is complete?
4. Do you have anything else you would like to share?

# Supplementary File 2. COREQ (Consolidated criteria for reporting qualitative research) 32-item checklist

Developed from:

Tong A, Sainsbury P, Craig J. (2007). Consolidated criteria for reporting qualitative research (COREQ): a 32-item checklist for interviews and focus groups. *International Journal for Quality in Health Care, 19*(6), 349 – 357.

| Topic | Item No. | Guide Questions/Description | Reported on Page No. | Notes |
| --- | --- | --- | --- | --- |
| **Domain 1: Research team and reflexivity** | | | | |
| *Personal characteristics* | | | | |
| Interviewer/ facilitator | 1 | Which author/s conducted the interview or focus group? | 7 | An experienced qualitative analyst was the primary interviewer; The lead researcher was a secondary interviewer on a few focus groups; One trainee was a co-facilitator for 1 patient focus group, for learning purposes. |
| Credentials | 2 | What were the researcher’s credentials? e.g. PhD, MD | Title page |  |
| Occupation | 3 | What was their occupation at the time of the study? | Title page | Indicated by Faculty/School. |
| Gender | 4 | Was the researcher male or female? | Title page | Denoted by name. |
| Experience and training | 5 | What experience or training did the researcher have? | Title page |  |
| *Relationship with participants* | | | | |
| Relationship established | 6 | Was a relationship established prior to study commencement? | 7 | No prior relationship. |
| Participant knowledge of the interviewer | 7 | What did the participants know about the researcher? e.g. personal goals, reasons for doing the research | 6 | The lead researcher introduced herself when she provided 2 clinician information sessions on June 20 and 23, 2017.  All participant signed informed consent forms, which outlined reasons for undertaking the research. |
| Interviewer characteristics | 8 | What characteristics were reported about the interviewer/facilitator? e.g. Bias, assumptions, reasons and interests in the research topic | 8 | Interviewers engaged in reflexive field notes (which were used as data), attending to their biases and assumptions. Field notes provided further contextual data. |
| **Doman 2: Study design** | | | | |
| *Theoretical framework* | | | | |
| Methodological orientation and Theory | 9 | What methodological orientation was stated to underpin the study? e.g. grounded theory, discourse analysis, ethnography, phenomenology, content analysis | 6, 7 | Guided by interpretive description methodology and the Knowledge-to-Action Framework. |
| *Participant selection* | | | | |
| Sampling | 10 | How were participants selected? e.g. purposive, convenience, consecutive, snowball | 6 | Purposive sampling. |
| Method of approach | 11 | How were participants approached? e.g. face-to-face, telephone, mail, e-mail | 6 | Patients: Patients attending their regularly-scheduled clinic appointments at both sites were initially screened and invited to participate by a unit clerk or a nurse, and then followed-up by study staff present in waiting rooms.  Clinicians: Clinicians were invited via information sessions, posters, and emails from the department.  All those willing to participate in focus groups or interviews shared their emails. All clinicians were invited to the workshop through emails from the department. |
| Sample size | 12 | How many participants were in the study? | 8 | Patients: 42 patients and 3 caregivers in focus groups and interviews.  Clinicians: 52 unique clinician participants across focus groups, interviews, and workshops |
| Non-participation | 13 | How many people refused to participate or drop out? Reasons? | 8 | Refusal to participate in focus groups/interviews was not tracked. No one dropped out. |
| *Setting* | | | | |
| Setting of data collection | 14 | Where was the data collected? e.g. home, clinic, workplace | 6, 7 | Patients: Interviews or focus groups took place at the implementation site, at the university, or over the phone.    Clinicians: Rating-scale data was collected at the implementation site.  Workshops were provided at the implementation site or at the university.  Interviews or focus groups took place at the implementation site, at the university, or over the phone. |
| Presence of non-participants | 15 | Was anyone else present besides the participants and researchers? | Table 3 | One family member attended a focus group so that a patient could participate; the family member did not join in the focus group discussion.  Three caregivers attended a focus group to support a patient to attend. |
| Description of sample | 16 | What are the important characteristics of the sample? e.g. demographic data, date | Table 2 and 3 |  |
| *Data collection* | | | | |
| Interview guide | 17 | Were questions, prompts, guides provided by the authors? Was it pilot tested? | 7, 9  Supplementary File 1 | Guides were not provided to participants. However, detailed outlines of workshops were co-developed and reviewed by clinicians at focus groups.  Workshop materials were also co-developed with Patient Partners and the research team.  Copies of all workshop PowerPoints and supplementary materials were provided to all workshop attendees. |
| Repeat interviews | 18 | Were repeat interviews carried out? If yes, how many? | 8 | Patients:  There were 6 focus groups in Phase 1; 3 in Phase 2.  There were 7 interviews in Phase 1; 11 in Phase 2.  18 patients participated in Phase 1 and 2.  Clinicians:  There were 6 focus groups in Phase 1; none in Phase 2.  There was 1 interview in Phase 1; 16 interviews in Phase 2.  16 clinicians participated in Phase 1 and 2 (excluding workshops). |
| Audio/visual recording | 19 | Did the research use audio or visual recording to collect the data? | 7 | Audio recorded and transcribed verbatim |
| Field notes | 20 | Were field notes made during and/or after the interview or focus group? | 8 | Field notes were written after each interview, focus group, and workshop. |
| Duration | 21 | What was the duration of the interviews or focus group? | 7 | Clinician and patient focus groups were 45-60 minutes;  clinician and patient interviews were 30-90 minutes; workshops were 45-60 minutes. |
| Data saturation | 22 | Was data saturation discussed? | 7 | Saturation was achieved when no new categories were identified and when participants’ descriptions became repetitive. |
| Transcripts returned | 23 | Were transcripts returned to participants for comment and/or correction? | 7 | Transcripts were not returned. But all participants in Phase 1 were invited to participate in Phase 2 during workshop delivery at the implementation site. |
| **Domain 3: analysis and findings** | | | | |
| *Data analysis* | | | | |
| Number of data coders | 24 | How many data coders coded the data? | 7, 8 | One experienced qualitative analyst and one trainee coded the data under the supervision of the lead researcher. |
| Description of the coding tree | 25 | Did authors provide a description of the coding tree? | 7, 8 | Codebook was iteratively refined to arrive at NVivo “parent and child nodes” |
| Derivation of themes | 26 | Were themes identified in advance or derived from the data? | 7, 8 | Themes were identified both in advance (to inform workshop development), and iteratively derived from the data. |
| Software | 27 | What software, if applicable, was used to manage the data? | 7 | NVivo |
| Participant checking | 28 | Did participants provide feedback on the findings? | 9 | Findings from Phase 1 informed development and delivery of the clinician workshops.  All participants in Phase 1 were invited to participate in Phase 2.  Clinician participants gave feedback on the workshops during Phase 2 interviews.  In Phase 2, patient participants shared their insights on how clinicians were using their questionnaire responses. |
| *Reporting* | | | | |
| Quotations presented | 29 | Were participant quotations presented to illustrate the themes/findings? Was each quotation identified? e.g. participant number | 9, 10  Table 5 | Table 5 provides additional exemplar quotes. |
| Data and findings consistent | 30 | Was there consistency between the data presented and the findings? | 8, 9, 10  Table 5 |  |
| Clarity of major themes | 31 | Were major themes clearly presented in the findings? | 8 |  |
| Clarity of minor themes | 32 | Is there a description of diverse cases or discussion of minor themes? | 8  Table 5 | Differences between participant groups are detailed throughout results and discussion sections. Negative / alternative cases are presented in Table 5. |
